# The sputum microbiome is distinct between COPD and health, independent of smoking history

**DOI:** 10.1186/s12931-020-01448-3

**Published:** 2020-07-14

**Authors:** Koirobi Haldar, Leena George, Zhang Wang, Vijay Mistry, Mohammadali Yavari Ramsheh, Robert C. Free, Catherine John, Nicola F. Reeve, Bruce E. Miller, Ruth Tal-Singer, Adam J. Webb, Anthony J. Brookes, Martin D. Tobin, Dave Singh, Gavin C. Donaldson, Jadwiga A. Wedzicha, James R. Brown, Michael R. Barer, Christopher E. Brightling

**Affiliations:** 1 Institute for Lung Health, NIHR, BRC, Department of Respiratory Sciences, College of Life Sciences, University of Leicester, Leicester, LE1 7RH UK; 2 Institute of Ecological Science, School of Life Science, South China Normal University, Guangzhou, 510631 China; 3 Department of Health Sciences, NIHR, BRC, University of Leicester, Leicester, LE1 7RH UK; 4 Global Medical, GSK, Collegeville, PA 19426 USA; 5 Department of Genetics, University of Leicester, Leicester, LE1 7RH UK; 6 University of Manchester and University Hospital of South Manchester, Manchester, M23 9QZ UK; 7 National Heart and Lung Institute, Imperial College London, London, SW3 6NP UK; 8 Computational Biology, Human Genetics, Research and Development (R&D), GlaxoSmithKline (GSK), Collegeville, PA 19426 USA; 9 Institute for Lung Health, University Hospitals of Leicester, Groby Road, Leicester, LE3 9QP UK

**Keywords:** COPD, Healthy airway, Microbiome, *Haemophilus*, *Proteobacteria*

## Abstract

**Background:**

Airway bacterial dysbiosis is a feature of chronic obstructive pulmonary disease (COPD). However, there is limited comparative data of the lung microbiome between healthy smokers, non-smokers and COPD.

**Methods:**

We compared the 16S rRNA gene-based sputum microbiome generated from pair-ended Illumina sequencing of 124 healthy subjects (28 smokers and 96 non-smokers with normal lung function), with single stable samples from 218 COPD subjects collected from three UK clinical centres as part of the COPDMAP consortium.

**Results:**

In healthy subjects *Firmicutes*, *Bacteroidetes* and *Actinobacteria* were the major phyla constituting 88% of the total reads, and *Streptococcus*, *Veillonella, Prevotella*, *Actinomyces* and *Rothia* were the dominant genera. *Haemophilus* formed only 3% of the healthy microbiome. In contrast, *Proteobacteria* was the most dominant phylum accounting for 50% of the microbiome in COPD subjects, with *Haemophilus* and *Moraxella* at genus level contributing 25 and 3% respectively. There were no differences in the microbiome profile within healthy and COPD subgroups when stratified based on smoking history. Principal coordinate analysis on operational taxonomic units showed two distinct clusters, representative of healthy and COPD subjects (PERMANOVA, *p* = 0·001).

**Conclusion:**

The healthy and COPD sputum microbiomes are distinct and independent of smoking history. Our results underline the important role for *Gammaproteobacteria* in COPD.

## Background

Chronic obstructive pulmonary disease (COPD) is characterised by inflammation and irreversible airflow obstruction. Before the advent of culture-independent DNA profiling methods, the healthy lung was deemed a sterile niche while COPD samples would frequently culture *Haemophilus influenzae, Streptococcus pneumoniae, Moraxella catarrhalis* and *Pseudomonas aeruginosa.* It is considered that pathogenic bacteria gain a foothold in damaged airways contributing to further lung pathology through release of noxious bacterial products and provocation of host inflammation [1, 2].

In recent years, use of high-throughput 16S rRNA gene based sequencing has demonstrated that rich, complex bacterial communities exist in the airways of both health and COPD, with overlapping bacterial composition observed [3, 4]. In COPD aerobic, facultative and anaerobic organisms colonise the airways [3, 5], with *Proteobacteria* and *Firmicutes* being the two major phyla reported in the microbiome and *Haemophilus* and *Streptococcus,* the respective dominant genera [3, 4, 6, 7]. At exacerbation, shifts in bacterial composition, characterised by a relative increase in *Proteobacteria* that falls in response to antibiotics has been observed, suggesting an association with the aetiology of COPD exacerbations [8, 9]. Furthermore, the ratio of *Gammaproteobacteria* to *Firmicutes* identifies the sub-group with dynamic changes in their microbiome during exacerbation, suggesting a potential use of this ratio as a biomarker for targeting antimicrobial treatment [10].

While *Proteobacteria* have been associated with COPD exacerbation events, the role of the microbiome in the stable state and important differences in composition with health are unclear [3, 4, 6]. Furthermore, changes in the microbiome that may associate with development of COPD in smokers are unclear. Differences have been shown in the nasal and oropharyngeal microbiome between smokers and non-smokers [11] but microbiome data from healthy smokers and non-smokers is limited making the findings inconclusive to contextualize the pathological basis of the observations in COPD.

To address this, we have used sputum collected from a substantial number of well characterised healthy volunteers to investigate the impact of smoking on healthy lower airway microbiome; to explore if there are differences in microbiome between health and COPD and its association with smoking in health.

## Methods

### Subjects and study samples

Healthy volunteers (*n* = 251) (excluded participants with asthma, COPD or bronchiectasis) were selected from the Extended Cohort for E-health, Environment and DNA (EXCEED) cohort and assessed at a single centre, Glenfield Hospital, Leicester. Participants with ≥10 pack year (PY) smoking were grouped as healthy smokers and remaining as occasional / never smokers (< 10 PY) [12]. Participant demographics and clinical characteristics including lung function, blood and sputum cell differentials were recorded. The comparator COPD group comprised of a single stable (non-exacerbation) visit sputum collected from 218 subjects (included ex- and current smokers) at three centres, Leicester, London and Manchester, as part of the COPDMAP consortium (www.copdmap.org; NCT01620645) [13]. All the participants in both studies had a minimum of 6 weeks antibiotic free period before their sample collection. Sputum induction was undertaken if an adequate spontaneous sample was not produced. In most of the healthy participants induced sputum was collected. Both cohort studies had ethical approval and all subjects gave written informed consent before the performance of any study-related assessments.

All patients provided written informed consent using protocols approved by the local Ethics Committees at each site (London- 11/L0/1630; Manchester- 10/H/1003/108; Leicester- 07/H0406/157).

### 16S rRNA gene sequencing

As a part of standard routine, for both studies, sputum plugs were separated from the salivary contents to minimise the oral bacterial contamination in samples. Samples with only salivary contents were not processed for microbial work. Sputum plugs, were stored (− 80 °C) and processed for high-throughput sequencing similar to the COPD cohort samples [13]. Briefly, bacterial genomic DNA was extracted from the homogenised (0.1% dithiothreitol) plugs using the lysozyme-based lysis procedure from Qiagen DNA Mini kit (Qiagen, CA, USA) as per manufacturer’s protocol. Out of 251 samples only 137 had adequate DNA concentration for microbiome analysis. Amplicon library was generated utilizing 28 PCR cycles and targeting the V4 hypervariable region of the 16S rRNA gene with 515F: 5′ GTGCCAGCMGCCGCGGTAA3’, 806R: 5’GGACTACHVGGGTWTCTAAT3’ primers, including Illumina sequencing adapters and a 12 bp Golay barcode sequence attached to forward primer. Pair-ended sequencing was performed using multiplex libraries on the Illumina MiSeq platform. Sequencing run included a commercial mock community DNA (ZymoBIOMICS microbial DNA standard) as a positive control and DNA extraction negative control (each batch of healthy sample DNA extraction included a DNA extraction negative control and a single pooled aliquot was prepared from all of these controls for sequencing) and PCR negative control for reagent contamination check. PCR negative controls didn’t produce any reads and DNA extraction negative control only produced 8 raw reads and therefore were not processed further.

COPDMAP single stable samples, utilized here for comparative analysis with healthy samples, had their sequence data generated as part of that study along with appropriate sequencing controls [13] and the sequence data are deposited at the National Centre for Biotechnology Information Sequence Read Archive (SRP102480).

### Microbiome analysis

Reads were processed using QIIME pipeline version 1.9.1 [14] after adaptor trimming and removing low quality reads with Trimmomatic 0.36 [14, 15]. Pair-ended sequences were joined using fastq-join with a minimum 10 bp overlap [16]. The joined sequences were filtered with a Phred score ≥ 20 and processed to remove contaminating sequences and chimeras using UCHIME [17]. Sequence reads are deposited at the National Centre for Biotechnology Information (SRA accession: PRJNA491861) https://www.ncbi.nlm.nih.gov/bioproject/PRJNA491861/.

Based on rarefaction curves, healthy and COPDMAP samples, were normalised to 11,000 reads sequencing depth leaving 124 samples in healthy and 218 samples in COPDMAP for microbiome analysis. Operational taxonomic units were generated at 97% sequence identity using close reference (OTU) method and Greengenes database (version 13_8) and assigned taxonomic identities with the RDP classifier [18]. Alpha diversity index was generated based on the number of OTUs (observed_OTUs and Chao1-richness measure) and their distribution (Shannon index- diversity measure) within a sample. Beta diversity index was based on weighted UniFrac distance measure [19] (phylogenetic distance-based) between OTUs present in each sample and visualized using PCoA plots.

### Microbial function prediction through 16S rRNA gene sequences

Predictive microbial functional profile was generated using the PICRUSt software (v1.0.0) which infers the pathway content of the microbiome by assigning bacterial functional genes for the OTUs, normalised for 16S rRNA gene copy number, using the Kyoto Encyclopaedia of Genes and Genomes (KEGG) database [20]. Statistical analysis and visualisation plots were generated using Statistical Analysis of Metagenomic Profiles (STAMP) software [21].

### Statistical analysis

Univariate statistical analyses were performed using GraphPad Prism (Version 7, San Diego, CA). Parametric and nonparametric data are presented as mean (SEM) and median (interquartile range) respectively. Most of the dataset did not meet the normal distribution criteria (Shapiro-wilk test). Therefore, non-parametric Mann-Whitney test and Kruskal-Wallis (KW) test were performed for between-group comparison of two or greater than two groups respectively. For the KW test, Dunn’s multiple comparison test was used to determine statistical significance of pair-wise comparisons. For parametric data, equivalent parametric statistical tests were performed. For categorical data, Chi-square test was performed.

PERMANOVA [22] was performed to test if the overall healthy microbiome was different to COPD using weighted UniFrac distance measure as input. The individual OTUs contributing to differences in health and COPD were identified by performing a non-parametric group-significance test and a corrected *p*-value of ≤0.05 for multiple testing conditions was considered statistically significant. To rank the discriminating taxonomic groups between health and COPD, linear discriminant analysis (LDA) effect size (LEfSe) analysis was performed [23], which detects taxonomies with differential abundance (*p* ≤ 0.05) using the KW test and then assigns a LDA based effect size score. A threshold of ≥3.6 LDA score was used instead of default value of 2 to feature the most discriminant bacterial groups [24] . To determine if any individual microbiome constituents were associated with any subject metadata, MaAsLin was performed [25] and Benjamini-Hochberg (FDR) corrected *q* < 0.05 (FDR-adjusted *P* value) for multiple comparisons was considered statistically significant.

## Results

Table 1 summarizes the demographics and clinical features of the healthy and COPD cohorts (refer to ‘Methods’ section for more details on both the cohort selection criteria).

**Table 1:** Healthy and COPD subject characteristics

	Healthy control (*n* = 124)	Healthy control PY ≥10 (*n* = 28)	Healthy control PY < 10 (*n* = 96)	COPD (*n* = 218)	*p*-value*
Gender female (n)	73	15	58	60	< 0·0001
Age (years)	61 (54, 67)	65 (58, 69)	59 (53, 67)	69 (64,74)	< 0·0001
BMI	27·7 (24·3, 31·2)	29·3 (25·8, 34·4)	27·3 (24·2, 29·9)	26·7 (23·32,30·06)	0·0325
Smoking history (Pack years)	0 (0, 8)	29 (17·3, 45·8)	0 (0,0)	46 (34,64)	< 0·0001
MRC Dyspnoea scale	0 (0, 1)	0 (0, 1)	0 (0, 1)	2 (1,2)	< 0·0001
VAS dyspnoea (mm scale)	0 (0, 2)	0 (0, 3.5)	0 (0, 2)	30 (15,49)	< 0·0001
Post BD FEV1 (L)	2·87 (2·43, 3·37)	2·82 (2·33, 3·24)	2·88 (2·46, 3·40)	1·45 (1·03,1·86)	< 0·0001
Post BD FEV1% Predicted	109 (100, 118)	105 (92, 118)	110 (100, 118)	57 (42, 69)	< 0·0001
Post BD FEV1/FVC %	80 (77, 83)	78 (75,82)	80 (78, 83)	51 (41,59)	< 0·0001
Blood White cell count (×10^9/L)	5·8 (4·9, 7·3)	6·4 (5·1, 7·5)	5·7 (4·8, 7·25)	7·2 (6·3,8·8)	< 0·0001
Blood Neutrophil count (× 10^9/L)	3·3 (2·7, 4·2)	3·8 (2·8, 4·3)	3·2 (2·6, 4·1)	4·7 (3·9, 5·7)	< 0·0001
Blood Eosinophil count (× 10^9/L)	0·14 (0·09, 0·23)	0·18 (0·11, 0·25)	0·14 (0·09, 0·22)	0·21 (0·14, 0·27)	< 0·0001
Sputum TCC (×10^6^/g)	2·4 (1·1, 4·2)	3·19 (1·6, 5·9)	2·2 (1, 3·6)	10·5 (4·7,13·0)	< 0·0001
Sputum eosinophils %	0·25 (0, 0·75)	0·25 (0·18, 2·38)	0·25 (0, 0·75)	0·75 (0,2)	0·0029
Sputum neutrophil (%)	60 (43, 74)	65 (42, 75)	56 (43, 73)	75 (40, 89)	0·0011

Data is expressed as median (IQR). *BMI* body mass index, *Sputum TCC* sputum total cell count, *FEV1* forced expiratory volume in 1 s, *Post BD FEV1* spirometry recording post bronchodilator; *FVC* forced vital capacity. * = represents Kruskal-Wallis test comparing between healthy (PY ≥10), healthy (PY < 10) and COPD

### Healthy sputum microbiome

**Fig. 1:**
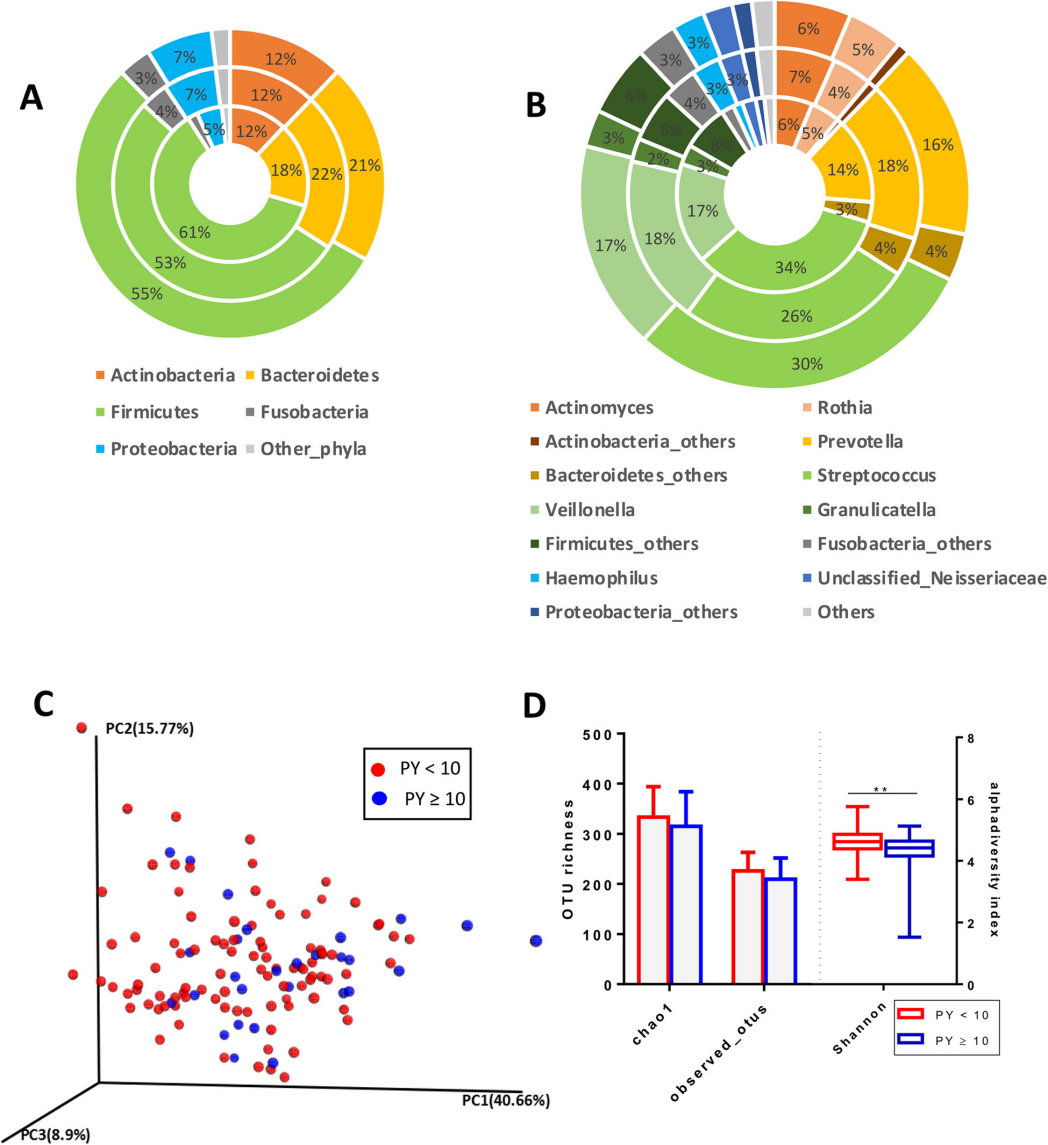
Microbiome profile of Healthy volunteers based on smoking pack year history. **a** Relative abundance of major phyla between all healthy (*n* = 124) represented in the outer ring followed by healthy < 10 PY smoking history subgroup (*n* = 96) in the middle ring and innermost ring representing healthy ≥10 PY history subgroup (*n* = 28). **b** Relative abundance of major genera between all healthy (*n* = 124) represented in the outer ring followed by healthy < 10 PY (*n* = 96) in the middle ring and innermost ring representing healthy ≥10 PY history (*n* = 28). **c** Principal coordinate analysis (PCoA) analysis of weighted unifrac distance measures relative to pack year history. **d** Alpha diversity indices comparison between. < 10 PY and ≥ 10 PY smoking sub-groups. Chao1 and observed_otus are represented as bar chart as mean and standard deviation; Shannon index is represented by box whisker plot showing median, interquartile range and minimum and maximum. **. *P* < 0.01

A total of 1424 OTUs at 97% sequence identity were observed in 124 healthy samples after rarefaction. Most OTUs belonged to *Firmicutes* (55% ± 13%) followed by *Bacteroidetes* (21% ± 11%) and *Actinobacteria* (12% ± 6%) phyla (Fig. 1a). *Streptococcus* (30% ± 13%) was the most abundant genus followed by *Veillonella* (17% ± 9%), *Prevotella* (16% ± 10%), *Actinomyces* (6% ± 5%), *Rothia* (5% ± 4%) and *Granulicatella* (3% ± 3%) (Fig. 1b). The *Proteobacteria* phylum constituted 7% (± 7%) of the bacterial community with *Haemophilus* (3% ± 5%) as its dominant genus.

Compared to participants with a smoking history of < 10 pack year (PY), the subgroup with ≥10PY showed a higher proportion of *Firmicutes* and a lower proportion of *Bacteroidetes* at phylum level (Fig. 1a); followed by higher *Streptococcus* and lower *Prevotella* at the genus level but these differences did not reach statistical significance (Fig. 1b). Beta diversity based principal coordinate analysis (PCoA) plots did not reveal distinct microbiome clusters for the two smoking subgroups (Fig. 1c). Shannon index was higher (*p* < 0.01) in healthy subjects with < 10 PY smoking history compared with ≥10 PY, suggesting a more diverse microbiome in the former (Fig. 1d) but there was no significant difference in observed_OTUs or Chao1 index.

### COPDMAP stable sputum microbiome

**Fig. 2:**
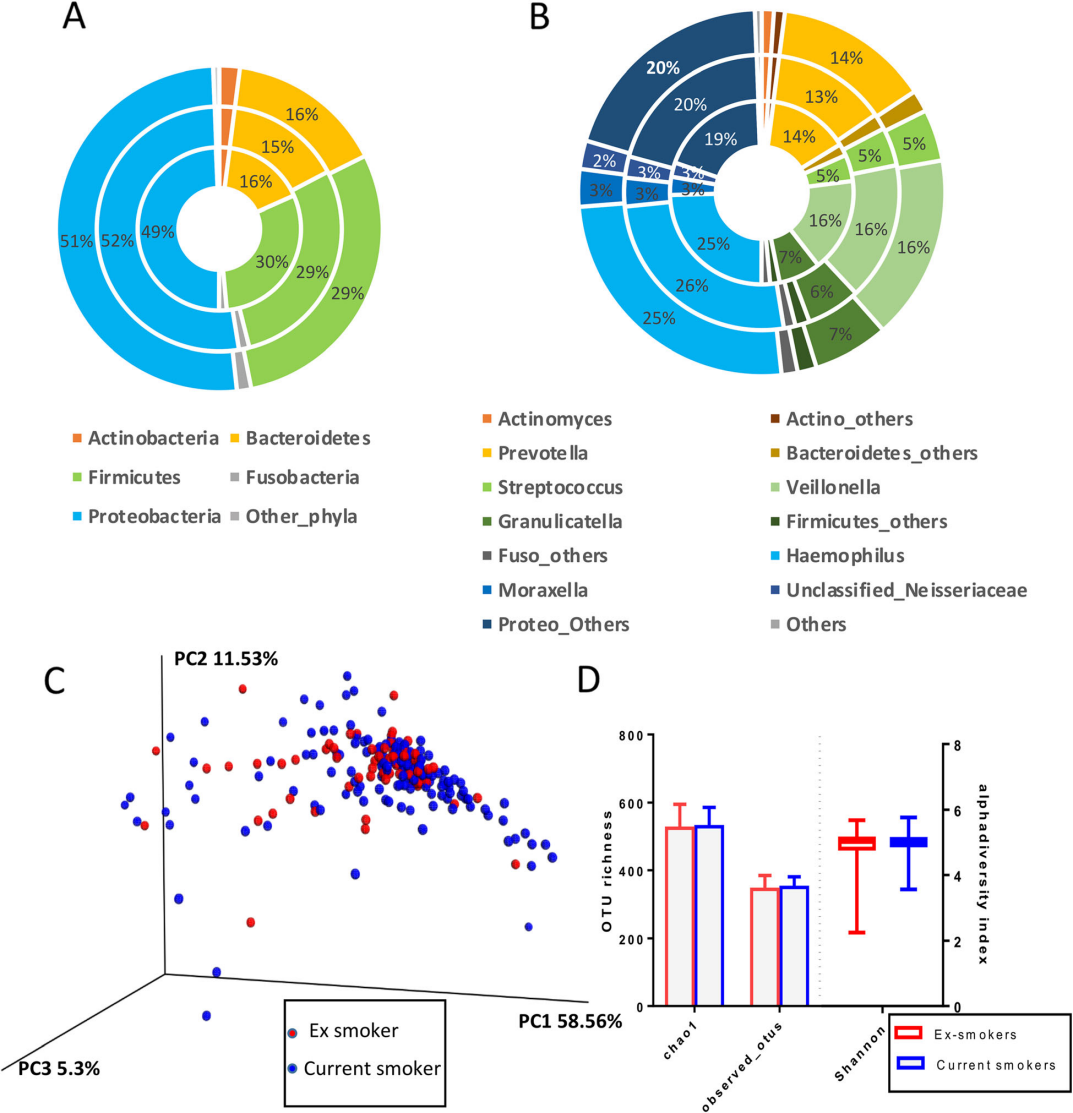
Microbiome profile of COPD subjects based on smoking pack year history. **a** Relative abundance of major phyla between all COPD (*n* = 218) represented in the outer ring followed by ex-smokers (*n* = 148) in the middle ring and innermost ring representing current smokers history (*n* = 70). **b** Relative abundance of major genera between all COPD (*n* = 218) represented in the outer ring followed by ex- smokers (*n* = 148) in the middle ring and innermost ring representing current smoker (*n* = 70). **c** PCoA analysis of weighted unifrac distance measures relative to pack year history. **d** Alpha diversity indices comparison between the two smoking groups

A total of 2329 OTUs at 97% sequence identity were observed in 124 stable COPD samples after rarefaction. The *Proteobacteria* (51% ± 12%) phylum constituted half of the COPD microbiome with *Haemophilus* (25% ± 8%) as its most abundant genus followed by *Erwinia* (7% ± 3%), *Cronobacter* (6% ± 2%) and *Moraxella* (3% ± 7%) (Fig. 2a and b). The two other abundant phyla were *Firmicutes* (29% ± 9%) and *Bacteroidetes* (16% ± 5%) dominated respectively by *Veillonella* (16% ± 9%), *Granulicatella* (7% ± 3%) and *Streptococcus* (5% ± 2%) and by *Prevotella* (14% ± 5%) at the genus level (Fig. 2a and b).

COPD ex-smokers and current smokers showed similar bacterial composition at phylum and genus level (Fig. 2a and b) and no significant difference in their microbial diversity measurements (Fig. 2c and d). There was no significant difference in the microbiome profile of the COPD samples from the three centres (efigure 1).

### Healthy vs COPD microbiome

**Fig. 3:**
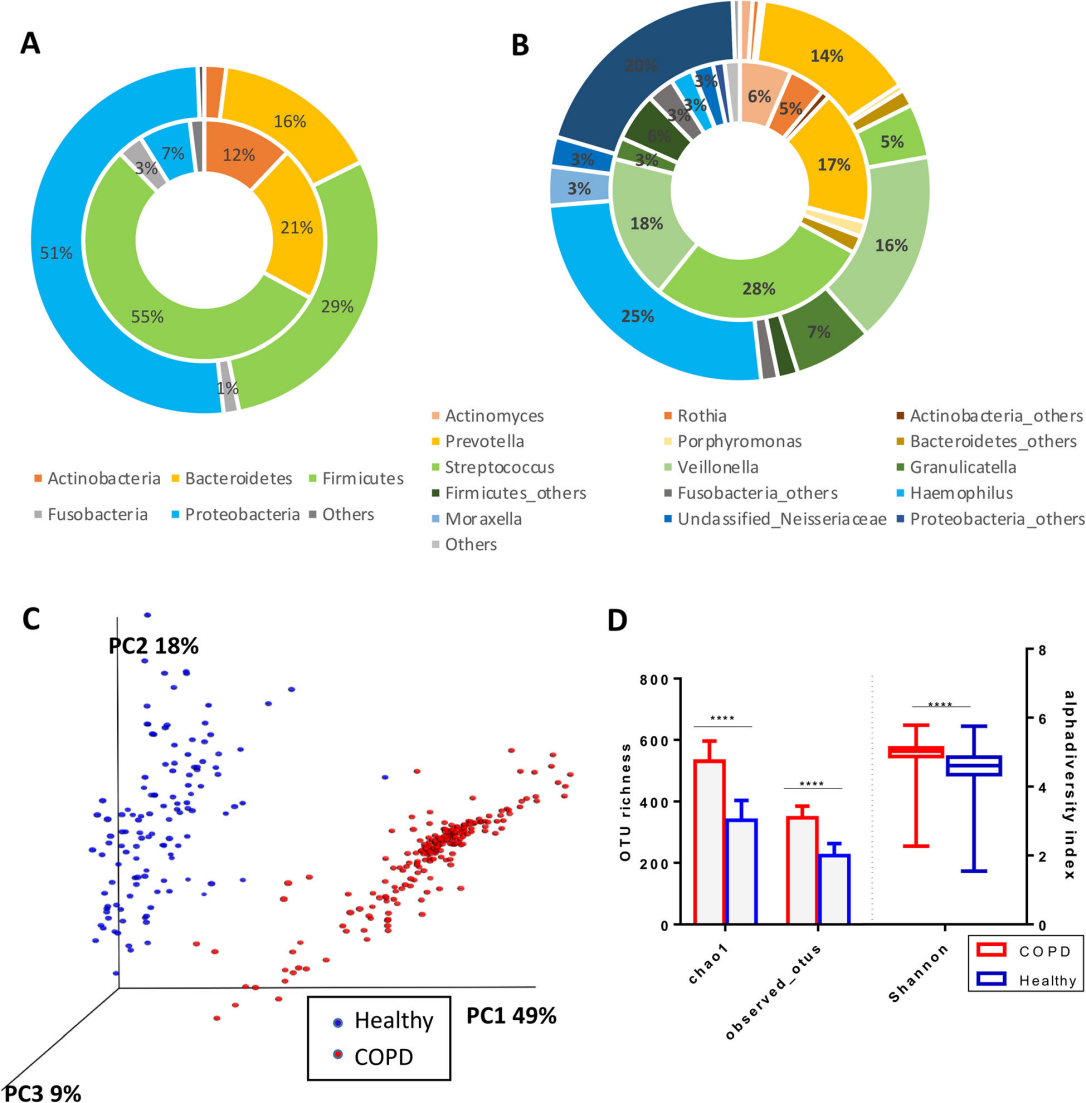
Comparison of Microbiome profile between Healthy and COPD. **a** Relative abundance of major phyla between COPD (*n* = 218) represented in the outer and inner ring representing healthy (*n* = 124). **b** Relative abundance of major genera between COPD (*n* = 218) represented in the outer ring and inner ring representing healthy volunteers (*n* = 28). **c** PCoA analysis of weighted unifrac distance measures between healthy and COPD subjects. **d** Alpha diversity indices comparison between Healthy and COPD subjects. ****, *P* < 0.00001

**Fig. 4:**
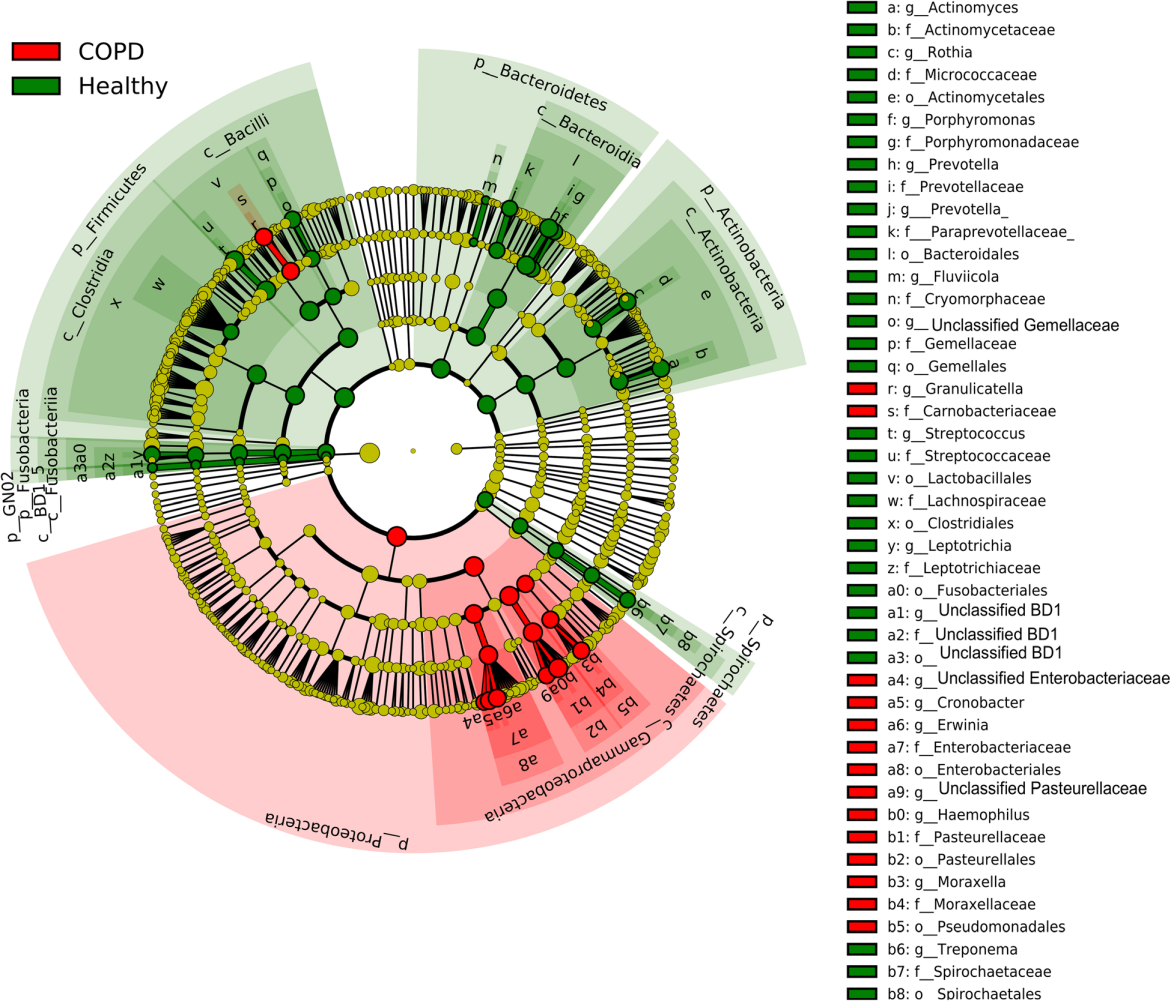
Bacterial groups distinguishing health and COPD microbiome. Each of the circles in the cladogram represent a bacterial taxa and each ring a taxonomy level starting with Kingdom (Archaea and Bacteria) in the innermost circle. Green coloured circles and zones represent bacterial taxa dominant in health and red in COPD. Circle sizes are correlated to bacterial abundance. Taxa level phylum (p_) and class (c_) are mentioned in the figure. Order (o_), Family (f_) and genus (g_) are abbreviated in the figure

In the healthy microbiome *Streptococcus* (28%) from the *Firmicutes* (55%) phylum was the predominant constituent, while *Haemophilus* (3%) from *Proteobacteria* (7%) was present at low levels. In contrast, for the COPD cohort *Haemophilus* (25%) was the most dominant genus with a low proportion of *Streptococcus* (5%) observed (Fig. 3a and b). PCoA analysis showed distinct clusters of healthy and COPD subjects with significant difference in their microbiome by permutation multivariate analysis of variance (PERMANOVA *p* = 0.01) (Fig. 2c). COPD samples had more OTUs identified compared to healthy individuals, with significantly higher alpha diversity indices (Fig. 3d). Linear discriminant effect size (LEfSe) analysis revealed a higher abundance of *Gammaproteobacteria* species and lower proportion of *Firmicutes*, *Bacteroidetes* and *Actinobacteria* taxa to be the major contributors in differentiating COPD from health (Fig. 4). OTUs differentiating the two groups are presented in Table 2.

**Table 2:** OTU groups that distinguish the Healthy and the COPD subjects

OTU	Taxonomy	Healthy_mean	COPD_mean	FDR_P	Bonferroni_P
579,608	Firmicutes|Bacilli|Lactobacillales|Streptococcaceae|**Streptococcus**	658	88	0.001	0·020
787,709	Actinobacteria|Actinobacteria|Actinomycetales|Actinomycetaceae|**Actinomyces**	363	64	0.001	0·020
1,078,207	Firmicutes|Bacilli|Lactobacillales|Streptococcaceae|**Streptococcus**	7	184	0.001	0·020
865,469	Proteobacteria|Gammaproteobacteria|Pasteurellales|Pasteurellaceae|**Haemophilus**	297	2150	0.001	0·020
585,419	Firmicutes|Clostridia|Clostridiales|Veillonellaceae|**Veillonella**	1611	1214	0.001	0·020
1,083,037	Proteobacteria|Gammaproteobacteria|Pseudomonadales|Moraxellaceae|**Moraxella**	12	345	0.001	0·020
968,954	Firmicutes|Bacilli|Lactobacillales|Streptococcaceae|**Streptococcus**	906	2	0.001	0·020
932,696	Proteobacteria|Gammaproteobacteria|Enterobacteriales|Enterobacteriaceae|**Erwinia**	0	782	0.001	0·020
579,924	Proteobacteria|Gammaproteobacteria|Pasteurellales|**Pasteurellaceae**|	2	210	0.001	0·020
1,083,194	Firmicutes|Bacilli|Lactobacillales|Streptococcaceae|**Streptococcus**	1290	5	0.001	0·020
1,027,587	Firmicutes|Bacilli|Lactobacillales|Carnobacteriaceae|**Granulicatella**	57	702	0.001	0·020
1,017,181	Actinobacteria|Actinobacteria|Actinomycetales|Micrococcaceae|**Rothia**	488	63	0.001	0·020
667,570	Proteobacteria|Gammaproteobacteria|Enterobacteriales|Enterobacteriaceae|**Cronobacter**	0	702	0.001	0·020
935,742	Bacteroidetes|Bacteroidia|Bacteroidales|Prevotellaceae|**Prevotella**	298	101	0.001	0·020
342,427	Firmicutes|Clostridia|Clostridiales|Veillonellaceae|**Veillonella**	44	284	0.001	0·020

Non-parametric t-test was performed on rarefied OTU table at 11,000 reds with OTUs that were observed in minimum 25% of the total number of samples and had a minimum contribution of 1% of the total reads

Multivariate analysis by linear models (MaAsLin) analysis detected no significant association either in COPD or in health between the bacterial groups and clinical characteristics related to smoking, lung function and symptom score.

**Fig. 5:**
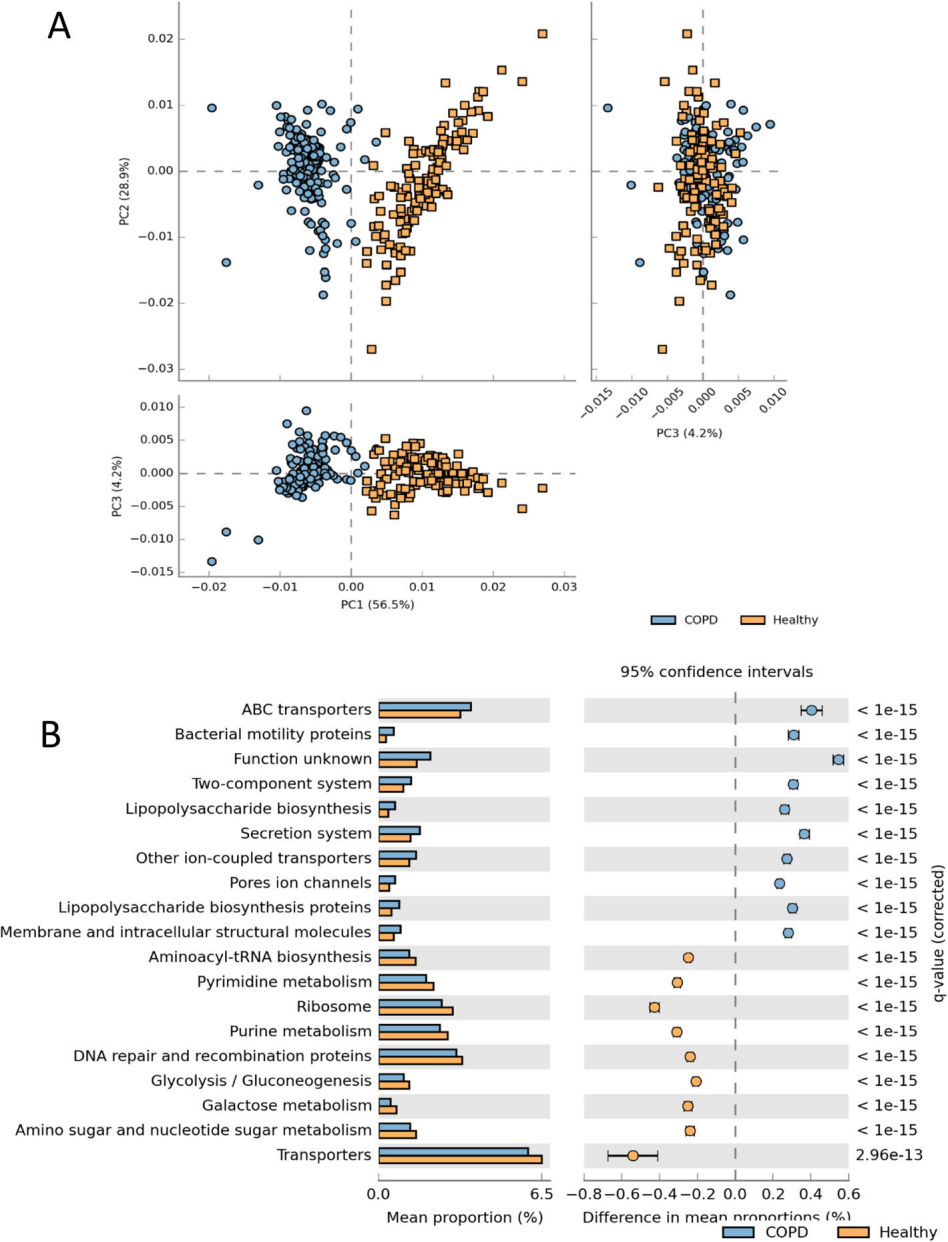
Predictive functional profiling shows distinct clustering of COPD and Healthy sputum microbiome. **a** PCA analysis of functional groups inferred from 16S rDNA microbial community. **b** lists the top 19 functional groups which were significantly different (*p* < 0.05, multiple comparison corrected) and had > 0.2% difference between COPD and healthy subjects

Phylogenetic Investigation of Communities by Reconstruction of Unobserved States (PICRUSt) analysis was performed to predict functional gene content from the 16S rRNA gene content. PCoA plots based on this analysis showed distinct clusters of COPD and health (Fig. 5a). Functional genes associated with Bacterial motility proteins, lipopolysaccharide biosynthesis, ABC transporters and secretion systems were in higher proportion in COPD while metabolic pathways were more abundant in healthy subjects (Fig. 5b).

## Discussion

Our study is the largest to compare the sputum microbiome between health and COPD and clear differences between these groups were identified. *Firmicutes*, *Bacteroidetes* and *Actinobacteria* comprised 88% of the sputum microbiome in healthy participants, with *Streptococcus, Prevotella* and *Veillonella* as the dominant genera. *Haemophilus,* the dominant genus in COPD was present in health at a significantly lower proportion. Healthy smokers with ≥10 PY smoking history showed a trend towards a higher ratio of *Streptococcus* to *Prevotella*.

Existing comparative respiratory microbiome data are divided, with some studies reporting an overlapping microbial composition between health and COPD [3, 4, 26] while others have shown the COPD microbiome to be distinct [5, 6, 27] . These discordant outcomes likely reflect the underlying heterogeneity in COPD groups and small sample sizes of healthy individuals (< 20), undermining the strength of these studies [3–6, 26, 27]. However, similar to our observation, higher levels of *Proteobacteria*, especially *Haemophilus,* in COPD [3–5] and relatively higher proportion of *Prevotella*, *Veillonella* and *Actinomyces species* in health have been observed [5, 6] but differences did not reach significance. Contrary to our observations, most studies have reported similar or a higher abundance of *Firmicutes* and especially *Streptococcus* in COPD compared with health [4, 6]. One reason for this might be that composition varies between samples depending upon the type of treatment received, disease severity and inflammation. Previous studies have shown association between very severe COPD and eosinophilic phenotypes with dominance of *Firmicutes*, while *Proteobacteria* are predominant in moderate COPD and the bacterial related phenotype [9, 28] . The COPD cohort analysed here was mainly of moderate-to-severe severity with high neutrophil counts.

Contrary to most studies, we found a higher alpha diversity in COPD compared to health [3, 6]. Although the COPD sample reads were reanalysed with the healthy at a normalised sequence depth, they were sequenced as part of COPDMAP study which involved a much larger sample size [13], including different disease stages, and this may have contributed higher COPD diversity. Moreover, our COPD cohort was older than the healthy group and from moderate to severe GOLD stage. Higher diversity has been associated with both increasing disease severity and age in COPD [4, 27, 29].

Although a strong association exists between smoking and both airway inflammation and COPD, the determinants for developing COPD in smokers are not clear. We hypothesised that smoking associated microbiome changes in health would help in understanding the role of microbes in transition from health towards COPD. Consistent with previous studies, we found no significant difference between the microbiome of smokers with ≥10PY history and the < 10 PY group [3, 4, 6, 30]. However, similar to Morris and colleagues [30], a trend towards lower proportions of both *Bacteroidetes* and *Proteobacteria* in smokers with ≥10PY history was observed, suggesting subtle effects of smoking on the airway microbiome. Other pathological factors may therefore be important in shaping the microbiome in COPD. Hypoxia and chronic systemic inflammation related factors, which are features of COPD, have been reported to be associated with the airway microbiome [31] and may be relevant to the differences observed in our COPD cohort.

PICRUSt analysis showed relatively higher lipopolysaccharide biosynthesis products in COPD. Lipopolysaccharides are present in the outer membrane of *Proteobacteria* and together with pathogen-associated molecular patterns, induce strong and damaging pro-inflammatory responses. In keeping with this, our previous study showed that sputum chemokine interleukin-8, known to play a key role in COPD inflammation, is positively correlated with *Haemophilus* and *Moraxella,* suggesting these bacteria trigger the excessive production of this chemokine [9]. Moreover, *Haemophilus* has been implicated in a dysbiotic role by co-inclusion of its related phylotypes and depletion of *Firmicutes*, *Bacteroidetes* and *Actinobacteria* that are involved in pathways for production of anti-inflammatory compounds [8, 9].

Although antibiotic treatment has been associated with suppression of *Proteobacteria* in COPD [8, 9], it is not true for all cases [10]. With the increasing urgency for effective antibiotic stewardship, research is needed to better understand the impact of both acute and long term antimicrobial therapy on the COPD microbiome. In this respect, alternate therapeutic strategies such as *H. influenzae* vaccination, or highly selective antimicrobial approaches such as phage therapy may effectively reverse some dysbiotic with prognostic benefit.

A limitation of this study is that the lung microbiome has been analysed from sputum samples which can be contaminated with the microbiome of the oropharynx. However, we emphasise that this effect will have been limited by sputum plug selection for the analysis. We did not perform longitudinal sampling to demonstrate reproducibility of the sputum microbiome over time in healthy participants. For COPD we have previously demonstrated that the sputum microbiome is comparable between time-points when sampling at their stable state [32]. The effects on the microbiome of using sputum induction as the predominant sampling technique in the healthy control group are also not known, but it is noteworthy that the predominant bacterial constituents of our healthy microbiome are consistent with the respiratory microbiome detected by investigating BAL and bronchial samples reported in previous studies [3, 6]. This suggests that our observations are robust and representative of the bacterial composition of the lung microbiome. A major incentive to work with sputum is its compatibility with routine clinical practice as any findings are therefore more readily translated into established care pathways. In this study we have not characterized the viral and fungal communities, and this will be important to understand their role in health and disease.

## Conclusions

In summary, clear and significant differences exist between the lung microbiome in health and COPD, with dysbiosis in COPD characterised by increased abundance of *Proteobacteria* especially *Haemophilus.* The changes observed in COPD are distinct from the microbiome in smokers without COPD, suggesting an association between airway damage and dysbiosis. The pathogenesis and pathological significance of dysbiosis in COPD remains unclear. Longitudinal studies are needed to determine whether, and to what extent, the onset and progression of COPD are attributable to an altered lung microbiome.

## Supplementary information

**Additional file 1.**

## Data Availability

Exceed healthy sample 16 rRNA gene sequence reads are deposited at the National Centre for Biotechnology Information (SRA accession: PRJNA491861) https://www.ncbi.nlm.nih.gov/bioproject/PRJNA491861/.COPDMAP 16S rRNA gene sequence data are deposited at the National Centre for Biotechnology Information Sequence Read Archive (SRP102480) and associated data available from (https://www.ncbi.nlm.nih.gov/search/all/?term=SRP102480).

## Abbreviations

COPD: Chronic obstructive pulmonary Disease
EXCEED: Extended Cohort for E-health, Environment and DNA
KW: Kruksal-Wallis
MaAsLin: Multivariate analysis by linear models
OTU: Operational taxonomic unit
PY: Pack year smoking history
PCoA: Principal coordinate analysis
PERMANOVA: Permutation multivariate analysis of variance
SEM: Standard error mean
LefSe: Linear discriminant effect size
