# Supplementary figures and images for: The sputum microbiome is distinct between COPD and health, independent of smoking history
Source: Respir Res. 2020 Jul 14;21:183. doi: 10.1186/s12931-020-01448-3 (PMC7362436; doi:10.1186/s12931-020-01448-3)

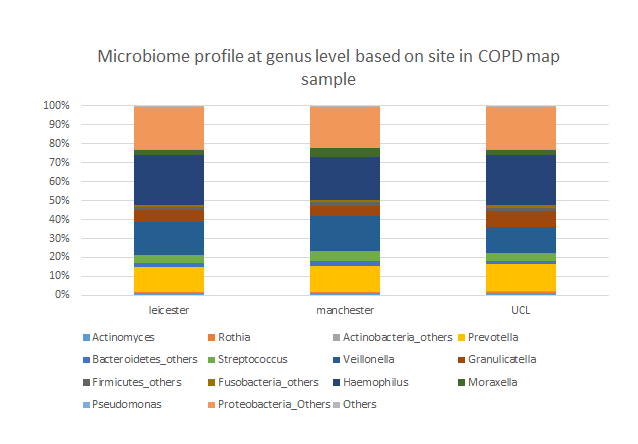

Supplement: Supplementary file 1 — Additional file 1. [file 12931_2020_1448_MOESM1_ESM.tif]
